# Supplementary material for: Content-rich biological network constructed by mining PubMed abstracts
Source: BMC Bioinformatics. 2004 Oct 8;5:147. doi: 10.1186/1471-2105-5-147 (PMC528731; doi:10.1186/1471-2105-5-147)
Supplement: Additional File 2 — The original results of the above study (non-essential files are deleted to keep the file size under the limit set by BMC bioinformatics). [file 1471-2105-5-147-S2.bz2 › chilibotAdditionalFile2/dip20/300ID7486683E1291/html/GRB2_PDGFRB.html]

 


 **GRB2** and **PDGFRB** 
  
Found 14 abstracts in PubMed, retrieved 14.  
 

 What does Google say? 
 PDF only 
| .edu only 

---

**Interactive relationship** (e.g. stimulation, inhibition, etc)

**Neutral relationship**- These data indicate that phosphorylation of SHPTP2 couples  **Grb2**  to PDGFR  [ **PDGFRB** ]  in vivo, providing a mechanism for Ras activation by PDGFR  [ **PDGFRB** ]  and for positive signaling via SHPTP2 and Csw.  Ref: 8041791 Proc Natl Acad Sci U S A, 1994
- Whereas the PDGFr  [ **PDGFRB** ]  associated with PtdIns 3 kinase, ras GAP, GRB 2  [ **GRB2** ] , and phospholipase C gamma, only GRB 2  [ **GRB2** ]  and PtdIns 3 kinase associated with IRS 1.  Ref: 7486683 Ann N Y Acad Sci, 1995
- When  **GRB2** , an adapter protein, was immunoprecipitated from treated VSMCs followed by Western blot analysis with anti phosphotyrosine, PDGFR  [ **PDGFRB** ]  alpha, and  **GRB2**  antibodies, respectively, phosphotyrosine positive staining was observed on PDGFR  [ **PDGFRB** ]  alpha bands of the same blot in stretch stressed VSMCs, supporting the mechanical stress induced activation of PDGFR  [ **PDGFRB** ]  alpha.  Ref: 9737716 FASEB J, 1998

**Non-interactive relationship** (e.g. studied together, co-existance, homology, etc.)

- Moreover, a mutant PDGFR  [ **PDGFRB** ]  lacking its SHPTP2 binding site displays markedly reduced  **Grb2**  binding.  Ref: 8041791 Proc Natl Acad Sci U S A, 1994
- In conclusion, our results demonstrate that Gi enables the PDGFR  [ **PDGFRB** ]  to signal more efficiently to p42 p44 MAPK, and this appears to be achieved through the regulation of c Src and Grb 2  [ **GRB2** ]  PI3K, which are intermediates in the p42 p44 MAPK cascade.  Ref: 9882612 Biochem J, 1999
- Some of the Shc associated phosphoproteins EGFR, PDGFR  [ **PDGFRB** ] , erbB 2, Met, bcr abl, H4 ret bound both the Shc and  **Grb2**  SH2 domains in vitro.  Ref: 7675449 Oncogene, 1995
- In a panel of mutant PDGF beta receptors, in which Tyr 716 and the previously known autophosphorylation sites were individually mutated, only PDGFR  [ **PDGFRB** ]  beta Y716F failed to bind  **GRB2** .  Ref: 7935391 Mol Cell Biol, 1994
- Tyr 1068 and 1086 from the C tail of EGFR, Tyr 684 from the kinase insert region of PDGFR  [ **PDGFRB** ] , and Tyr 895 from IRS 1 were confirmed as major binding sites for the  **Grb2**  SH2 domain.  Ref: 8621421 J Biol Chem, 1996
